# Women and global health: a personal view

**DOI:** 10.1017/gheg.2016.6

**Published:** 2016-06-15

**Authors:** J. A. Whitworth

**Affiliations:** Emeritus Professor Australian National University

**Keywords:** Global health, policy and society, underrepresented, women

Women have been recognised as playing a central role in global health over millennia. Hygieia in both Greek and Roman mythology was the goddess of good health, cleanliness and sanitation, and her sisters Panacea, Acesco and Laso were goddesses of remedy, healing and recuperation, respectively. Florence Nightingale became a cult figure during the Crimean War and was a key figure in social reforms designed to improve health care across all levels of society. She is credited as the founder of modern nursing. More recently, a few women have made it to the top in global health, e.g. at international level Gro Harlem Bruntland and now Margaret Chan as Directors General of WHO, and at national level Dame Sally Davies as Chief Medical Officer of the UK (I was the Australian equivalent in the late nineties).

## The problem

Despite their undoubted skills and expertise, women remain substantially underrepresented in leadership positions in global health. In Australia, and indeed most, women constitute more than half of health professionals, and biomedical scientists but are greatly underrepresented in senior roles. Why?

Perhaps the first thing to say is that this is part of a much more general phenomenon. This is not a problem confined to global health. Underrepresentation of women in leadership roles is ubiquitous. Women are few and far between as heads of state, as parliamentarians, as CEOs in both private and public sectors and in leadership roles generally. And by and large the root causes are the same.

Sheryl Sandberg, COO of Facebook, speaking on why we have too few women leaders pointed out that the choices around career are harder for women. She quoted data indicating that male married senior managers were twice as likely as female counterparts to have children. Thus for many women, career choices may limit lifestyle, most importantly the decision to have a family. Not all women can have it all. Men rarely are confronted with this often devastating choice. In all studies that have examined the question working women do more of the housework and more of the child care than their working male partners, and they are the ones expected to cope in a domestic emergency. Women who do opt to try to combine career and family often have to deal with a degree of social opprobrium and with guilt. The paucity of appropriate child care facilities reinforces the problem.

Sandberg also quoted data indicating women systematically underestimate their own abilities and capacities. This phenomenon is very familiar in academia. In my experience, and that of many others, women who are well qualified often doubt their readiness for promotion, whereas less qualified men usually have no such doubts. Women tend to be poor negotiators for their own interests: for example, they are often reticent to ask for salary increases or privileges, reluctant to put themselves forward, and reluctant to take credit, all of which inhibits advancement and contributes to their lack of recognition. In many societies, women are trained either consciously or unconsciously to be self-effacing and not to be seen as pushy. Behaviour that is entirely acceptable in men is often unacceptable in women.

Sandberg quotes findings that success and likeability are positively correlated for men but negatively for women. We have all seen behaviour in men described as assertive and strong, whereas in a woman that same behaviour is frequently labelled as aggressive and tough. Sandberg had a very revealing anecdote about giving a talk to a group of employees. A woman came to her afterwards and said ‘I learned something today. I learned I need to keep my hand up.’ As she told it, at the end of the talk Sandberg said she would take two more questions. After the two, all the women put their hands down, but most of the men left their hands up, and there were more questions, all from men. Sandberg realised that we are not good at recognising that men reach for opportunities more than women.

Sexual harassment is a further dimension. Recent stories about bullying and harassment in surgery and astronomy, and the tendency of institutions to turn a blind eye, are only the tip of the iceberg.

Just this year UNs Ban Ki-moon pointed out that ‘empowerment of the world's women is a global imperative’. Unfortunately many people do not accept this view, let alone know how to change it.

Some years ago, as Australian CMO, I was leader of the Australian delegation to the World Health Assembly. In my first year, there was an election for the WHO Director General position, and I was lobbied intensively by candidates and their supporters. One such function was a small dinner party I attended with ministers, officials and WHO staff. I was the only woman. During the evening I was asked my opinion of the candidates. I said I thought the two women in the field were outstanding. There was a short silence and then it was explained to me that the job was too big for a woman. I pointed out that one of the candidates had run a European country and the other a UN agency. It was politely suggested to me that as both women were grandmothers their domestic responsibilities would mean they could not undertake such a demanding role. The story had a happy ending with the election of Mrs Bruntland, but I am not sure these attitudes are much changed.

In any competitive field, small handicaps can make a huge difference. Think of the Olympics where fractions of a second can make the difference between success and failure. Given the professional handicaps of time out, domestic load and societal pressures and expectations, it is perhaps surprising that women do as well as they do. Along these same lines, Mark Toner who is chair of the Australian Academy of Technological Sciences and Engineering's Gender Equity Working Group reported that modelling of an organisation with initially gender parity at the lowest level showed a mere 1% bias against women in promotion decisions produced almost twice as many men as women in senior levels. Bias may be unconscious, but conscious bias is also alive and well.

Gender attitudes permeate all levels of society and start very young. Years ago, when my daughter was tiny, someone remarked to her that both her parents were doctors. ‘No’ she replied. ‘Daddy is a doctor and Mummy is a nurse’.

From a societal perspective, failure to use the skills and expertise of women is not just a matter of equity, but detrimental to productivity and a poor return on public investment in education and training.

## What are the solutions?

Initiatives that have been used or proposed to promote women include recognition that appointment committees and granting bodies need to judge people by years in the workforce rather than years since graduation, thus recognising time out for child birth and family commitments, and fellowships designed to assist workplace return after enforced time out, linking funding to gender equity programmes. Mark Toner has suggested best practice would be for a recruitment panel to discuss their own biases prior to seeing the candidates, to have descriptors of relevant biases to hand, and after interview to discuss how their own biases were addressed. If only…De-identifying the gender of applicants where possible might also help … a study reported in the Guardian found that for GitHub, one of the world's largest open source software communities, code written by women was more likely to be approved by peers than that written by men, but only when they didn't know it was written by a woman.

One promising initiative in Australia is SAGE…the Science in Australia Gender Equity project. This is an academic initiative aiming to ensure as many women as men take up leadership in science. The figures here reflect what seems to be generally true across both fields and countries…women are over half doctoral graduates but only 17% of senior academics and researchers, with consequent waste of expertise and community investment. The plan is to collect data on gender equity policies, practices and outcomes and identify areas for improvement.

Leaders, both men and women, need to be role models for female advancement through their recruitment and promotion strategies, work place flexibility and very importantly through their rhetoric. They need to mentor and encourage women. Sue Pond, Co-chair of SAGE has pointed out the need for male as well as female champions if we are to overturn entrenched gender inequality in our institutions.

Many people do recognise that denying women opportunity is not simply unfair, but very wasteful. Prime Minister Trudeau in Canada had no difficulty reaching gender parity in his cabinet…the hoary old chestnut that merit should be the ultimate criterion is shown for what it is…rationalisation of discriminatory practice.

However, it is clear that even where societies have legislated for gender equality, marked disparities remain. Cultural attitudes dating back millennia are hard to shift.

The Athena SWAN Charter in the UK is an important initiative to address gender inequalities in science with six principles: first, it requires commitment and action from everyone in an organisation, at all levels; second, changing cultures and attitudes; third, the need to examine the implications of absence of diversity at top levels; fourth, the need to address the high dropout rate of women in science; fifth, to recognise the negative consequences of short-term contracts for women, and sixth, that broader personal, societal, environmental and governmental factors, among others, must be considered.

Transparency is fundamental if we are to have greater equity…if organisations have to be accountable, e.g. for salary parity, and then behaviour improves.

In February, we had the first United Nations International Day of Women and Girls in Science. This is undoubtedly a step in the right direction but there is a long way to go.

To make progress we need recognition that there is a problem, that a paucity of women leaders is harmful to society and not just women; we need commitment to change at all levels; we need transparency around choices and decision making; we need mentors and role models and we need affirmative actions to overcome the unconscious biases present in even the well intentioned.

In my lifetime, we have made great strides forward in gender equity. I believe we will do much more in my daughter's lifetime. Global health will be all the stronger for it.

